# On the Hygienic Influence of Cutting the Hair

**Published:** 1848-12

**Authors:** 


*On the Hygienic Influence of Cutting the Hair.*—Medical men are occasionally asked whether it is proper to cut the patient's hair; whether, in fact, this operation has any influence upon the health. M. Fredericque resolves the question by giving the following illustration:—

A little girl, aged three, of good health in general, had her hair grown excessively long in the course of a few months. She was a beautiful child, but had latterly wasted without any apparent cause, becoming dull and apathetic, losing her appetite and strength without any organic lesion being discernible. There was an anæmic bruit in the carotid. She was placed upon a tonic regimen, with chalybeates, but without deriving material benefit, until her hair was cut short, at the suggestion of a friend, from which time she rapidly gained strength.

It would appear from this case that the economy had suffered a loss in the expenditure of blood necessary for the secretion of the abundant crop of hair. M. Fredericque considers that it is the formation of the coloring matter which chiefly exhausts the blood, as this is formed at the expense of the hæmatosine.—*Annales de Societe d'Emulation; Revue Medico-Chirurgicale.*

